# Bioactive Glass-Ceramic Scaffolds from Novel ‘Inorganic Gel Casting’ and Sinter-Crystallization

**DOI:** 10.3390/ma10020171

**Published:** 2017-02-13

**Authors:** Hamada Elsayed, Acacio Rincón Romero, Letizia Ferroni, Chiara Gardin, Barbara Zavan, Enrico Bernardo

**Affiliations:** 1 Department of Industrial Engineering, University of Padova, Via Marzolo 9, 35131 Padova, Italy; hamadasaidabdelwahab.elsayed@unipd.it (H.E.); acacio.rinconromero@unipd.it (A.R.R.); 2 Ceramics Department, National Research Centre, El-Bohous Street, Cairo 12622, Egypt; 3 Department of Biomedical Sciences, Universiy of Padova, via Ugo Bassi 58/B, 35131 Padova, Italy; letizia.ferroni@unipd.it (L.F.); chiara.gardin@unipd.it (C.G.); barbara.zavan@unipd.it (B.Z.)

**Keywords:** alkali activation, gel casting, bioactivity, wollastonite, diopside, glass-ceramics

## Abstract

Highly porous wollastonite-diopside glass-ceramics have been successfully obtained by a new gel-casting technique. The gelation of an aqueous slurry of glass powders was not achieved according to the polymerization of an organic monomer, but as the result of alkali activation. The alkali activation of a Ca-Mg silicate glass (with a composition close to 50 mol % wollastonite—50 mol % diopside, with minor amounts of Na_2_O and P_2_O_5_) allowed for the obtainment of well-dispersed concentrated suspensions, undergoing progressive hardening by curing at low temperature (40 °C), owing to the formation of a C–S–H (calcium silicate hydrate) gel. An extensive direct foaming was achieved by vigorous mechanical stirring of partially gelified suspensions, comprising also a surfactant. The open-celled structure resulting from mechanical foaming could be ‘frozen’ by the subsequent sintering treatment, at 900–1000 °C, causing substantial crystallization. A total porosity exceeding 80%, comprising both well-interconnected macro-pores and micro-pores on cell walls, was accompanied by an excellent compressive strength, even above 5 MPa.

## 1. Introduction

In the field of bioceramics, those based on Ca-silicates and Ca-Mg silicates have recently received a growing interest for their bioactivity properties, according to their ability to stimulate body tissues to repair themselves, in particular for bone ingrowth [1,2,3,4,5,6,7]. Many experiences actually concern glass-ceramics from the controlled crystallization of glasses belonging to the CaO-MgO-SiO_2_ system with B_2_O_3_, Na_2_O, CaF_2_, and P_2_O_5_ additives [6,8,9,10]. While additives may lead to the formation of additional phases, such as fluorapatite [10], the main oxide leads to ternary silicates, such as akermanite, Ca_2_MgSi_2_O_7_, as well as mixtures of binary and ternary silicates, e.g., wollastonite, CaSiO_3_, coupled with diopside, CaMgSi_2_O_6_.

Bioactive glass-ceramics, instead of bioglasses, generally offer the possibility to maximize the mechanical properties of highly porous, open-celled foams. The open-celled morphology is fundamental, in bone-tissue applications, for cell ingrowth and vascularization [11], but it generally limits the mechanical strength. For an ideally open-celled solid, the crushing strength σ_c_, according to the well-recognized Gibson-Ashby model [12], depends largely on the relative density (ratio between geometric and true densities, ρ_rel_), as follows:

$$\sigma_{c}\;\propto \sigma_{\mathrm{bend}}\cdot {\left(\rho_{\mathrm{rel}}\right)}^{1.5}$$

where σ_bend_ is the bending strength of the solid phase. High porosities, and consequently low relative densities, determine a severe ‘downscaling’ of strength, given the exponential correlation. The enhancement of the strength of the solid phase, by crystallization, may provide a valid compensation [13].

The strength of the solid phase, however, is not simply tuned by the degree of crystallization. On the contrary, it may depend on the ‘quality’ of the manufacturing process. More precisely, many glass and glass-ceramic foams are produced by the classical replica method, i.e., by the coating of polyurethane sacrificial templates with glass slurries [13,14,15]. According to this method, glass undergoes viscous flow sintering, with the thermal treatment, along with burn-out of the substrate. The mechanical strength of the final cellular material can be negatively influenced by the formation of hollow struts, by the sintering of glass around former polymeric struts, so that a careful control of the sintering conditions is needed; the viscous flow of glass, under optimized conditions, may lead to the removal of the internal porosity [16].

A strategy to obtain highly porous foamed scaffolds with generally denser struts is that of gel-casting. The method actually yields combinations of macro- and micro-porosity; while macropores (typically >500 µm), like in replica-derived scaffolds, favor cell ingrowth and vascularization, micro-pores (and also nano-pores) in the cell walls favor cell attachment [11,15]. Starting from the early 2000s, gel casting has been widely applied to sol-gel formulations [15,17]; air bubbles may be incorporated by mechanical stirring of solutions (‘direct foaming’) at the early stages of gelification (sol state), with the help of surfactants, and kept by the progressive hardening (transition to the gel state). Intensive research, especially conducted by Jones and Hench [18,19,20], has demonstrated that the pore architecture can be tuned, operating on the many variables of sol-gel processing, such as chemistry of both glass and surfactants. In parallel, gel-casting has been applied even to suspensions of glass powders, subjected to gelification according to the addition of specific organic agents (monomers, cross-linkers, and catalysts) [21,22]. Highly porous gelified suspensions are converted into glass scaffolds by a sintering treatment, causing also the burn-out of any organic fraction.

The present paper is essentially aimed at presenting a new approach to glass-ceramic foams implying a revision of the gel casting process for direct foaming, starting from alkali activation of glass powders, followed by sinter-crystallization, i.e., viscous flow sintering of glass with concurrent crystallization. The alkali-activation is actually receiving growing interest in the fields of ceramics. Usual alkali-activated materials, generally known as “geopolymers”, are produced through the reaction of an alumino-silicate with an alkaline compound, which is typically a concentrated aqueous solution of alkali hydroxide or silicate [23]. These raw materials yield a ‘zeolite-like’ gel, consisting of a continuous, three-dimensional alumino-silicate network, amorphous or crystalline [23]. The network features the bridging of [SiO_4_] and [AlO_4_] tetrahedra, the latter being formed by the presence of alkali ions in the surrounding spaces, for the charge compensation. The alkali ions remain substantially ‘trapped’ in the alumino-silicate network, for an optimum Al_2_O_3_/SiO_2_ balance in the raw materials, with the achievement of chemically stable products. A gel is formed, in any case, also from formulations with different Al_2_O_3_/SiO_2_ balances; as an example, CaO-rich formulations do not yield a ‘zeolite-like’ gel, but provide a condensation product that could be termed ‘tobermorite-like’ gel, given the analogy with the products of cement hydration [23]. As a consequence, the term ‘inorganic polymer’ may sound more appropriate to identify the products, being independent from the structure of the gel [23,24].

The concept of alkali activation and ‘inorganic polymerization’ may be applied also to glasses as raw materials. Glasses with engineered chemical composition (alumino-silicate glasses) can be used as precursors for geopolymer-like materials [25,26,27] to be used as new binders for the building industry, according to the formation of sodium alumino-silicate hydrate (N–A–S–H) and calcium alumino-silicate hydrate (C–A–S–H) gels. With proper molecular balances between different oxides, both strength and chemical stability are optimized. Recycled glass can be used as a component of mixtures yielding geopolymers [28,29,30]; if a zeolite-like gel is not the target, even only common soda lime-glass cullet, activated with sodium or potassium hydroxide solutions, can be used. The so-obtained ‘glass-based mortars’, cured at 40–60 °C, achieve good mechanical strength (compressive strength of 50 MPa), but limited durability [31].

The present investigation recovers the idea of glass-based mortar, as an intermediate product for glass foams, to be stabilized by thermal treatment, as recently found for soda-lime glass [32]. Suspensions of fine glass powders, at the early stages of gelification, may trap air by intensive mechanical stirring with the help of a surfactant, in analogy with the processing of highly porous geopolymers [33]. The cellular structure is stabilized first by the progression of gelification, at low temperature, and then by viscous flow sintering, with concurrent crystallization, upon firing. The crystallization of glass, besides enhancing the mechanical properties, is intended to impede excessive viscous flow, that could lead to the collapse of the cellular structure, in analogy with previous experiences [34].

## 2. Experimental Procedure

### 2.1. Starting Glass

The reference material for the present investigation consisted of a glass belonging to the CaO-MgO-SiO_2_ system. The overall composition (SiO_2_: 51.7 wt %; CaO: 32.1%; MgO: 11.5%; Na_2_O: 2.2%; P_2_O_5_: 2.5%) corresponds to a CaO:MgO:SiO_2_ molar ratio equal to 2:1:3, theoretically leading to 50 mol % wollastonite (W, CaO·SiO_2_) and 50% diopside (D, CaO·MgO·2SiO_2_), so that it will be later referred to as W-D glass. The glass was produced from pure minerals and chemicals (silica, dolomite, calcium carbonate—all in powders <10 µm, Industrie Bitossi, Vinci, Italy—and sodium phosphate—sodium pyrophosphate, Na_4_P_2_O_7_, Sigma-Aldrich, Gillingham, UK), by melting in a platinum crucible at a temperature of 1400 °C (heating rate of 10 °C/min).

The mixture led to a homogeneous glass, despite the short holding time (15 min at 1400 °C), that was suddenly cooled by direct pouring on a cold metal plate. The glass fragments were easily reduced into fine powders by ball milling and later manually sieved; only the particles with a diameter below 75 µm were kept.

### 2.2. Preparation and Microstructural Characterization of Foams

**Figure 1:**
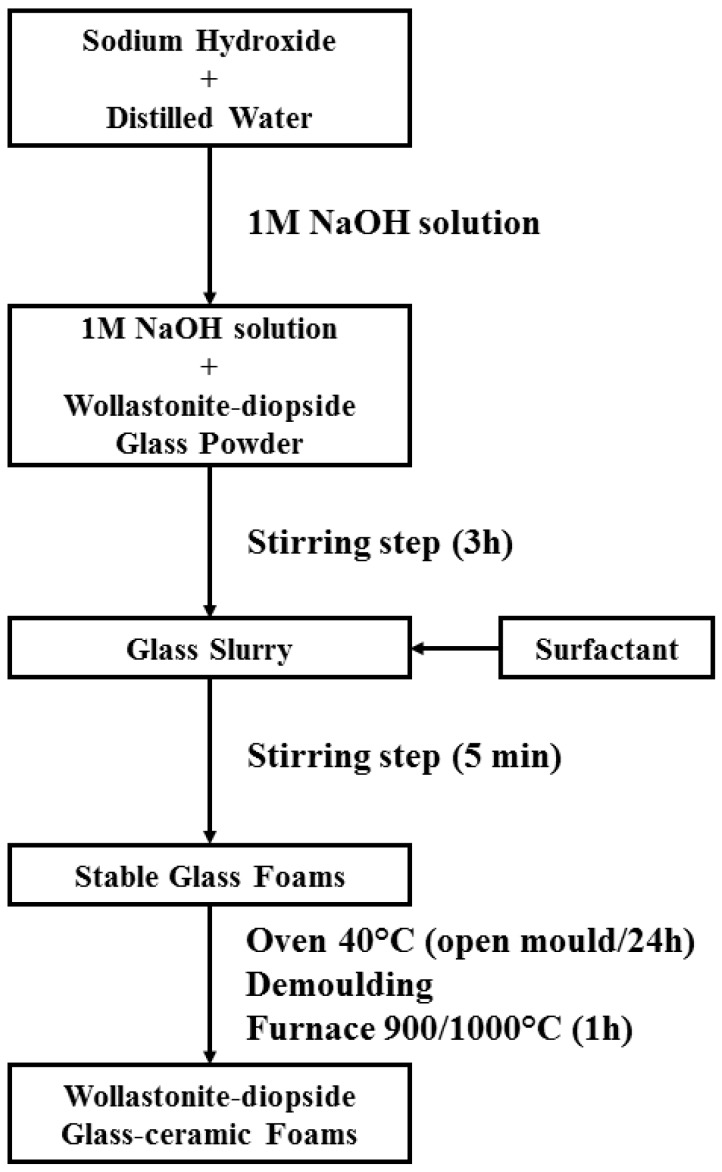
Flow chart of the W-D glass foams processing using the alkaline activation and gel casting.

W-D glass fine powders were introduced in an aqueous solution containing 1 M NaOH (reagent grade, Sigma-Aldrich), for a solid loading of 60 and 65 wt %. The glass powders were subjected to alkaline attack for 3 h, under low speed mechanical stirring (500 rpm). After alkaline activation, the obtained suspensions of partially dissolved glass powders were cast in several polystyrene cylindrical moulds (60 mm diameter) and then added with 4 wt % Triton X-100 (polyoxyethylene octyl phenyl ether—C_14_H_22_O(C_2_H_4_O)_n_, *n* = 9–10, Sigma-Aldrich), a non-ionic surfactant that does not interfere with ceramic dispersions [35]. The mixtures were foamed by vigorous mechanical mixing (2000 rpm), for 5 min and later left at 40 °C for 24 h in order to complete the gelation before demolding. It should be noted that the foamed samples were easily handled, after demolding, without any heat treatment applied. Finally, hardened foams were fired at 900–1000 °C for 1 h with a heating rate of 2 and 5 °C/min. Figure 1 shows the flowchart of process used for fabricating wollasonite-diopside (W-D) glass-ceramic foams.

W-D glass powders and foamed gels were subjected to thermogravimetric analysis (TGA, STA409, Netzsch Gerätebau GmbH, Selb, Germany) and Fourier-transform infrared spectroscopy (FTIR, FTIR model 2000, Perkin Elmer, Waltham, MA, USA). The crystalline phases were identified by means of X-ray diffraction on powdered samples (XRD; Bruker D8 Advance, Bruker AXS GmbH, Karlsruhe, Germany), supported by data from PDF-2 database (ICDD-International Centre for Diffraction Data, Newtown Square, PA, USA) and Match! program package (Crystal Impact GbR, Bonn, Germany).

The bulk density of the foams was determined from the weight-to-volume ratio, using a caliper and a digital balance. The true density of the samples was measured by means of a gas pycnometer (Micromeritics AccuPyc 1330, Norcross, GA, USA), operating with He gas on finely milled samples. The compressive strength of foams was measured at room temperature, by means of an Instron 1121 UTM (Instron, Danvers, MA, USA) operating with a cross-head speed of 1 mm/min. Each data point represents the average value of 5 to 10 individual tests. 

Microstructural characterizations were performed by optical stereomicroscopy (AxioCam ERc 5s Microscope Camera, Carl Zeiss Microscopy, Thornwood, NY, USA) and scanning electron microscopy (SEM) equipped with energy dispersive spectroscopy (EDS) (FEI Quanta 200 ESEM, FEI, Hillsboro, OR, USA).

### 2.3. Assessment of the In Vitro Bioactivity and Cell Culture Test

For cell culture studies, samples were cut to 10 × 10 × 5 mm^3^ and sterilized by autoclaving at 121 °C for 20 min. Samples were then fixed to 48-well plates. Normal human adult dermal fibroblasts (ATCC^®^-PCS-201-012™; American Type Culture Collection, Manassas, VA, USA) were seeded at a density of 4 × 10^5^ cells/piece in cDMEM, which consisted of Dulbecco’s Modified Eagle Medium (DMEM) (Lonza S.r.l., Milano, Italy), supplemented with 10 vol % Fetal Bovine Serum (FBS) (Bidachem-Spa, Milano, Italy) and 1 vol % Penicillin/Streptomycin (P/S) (EuroClone, Milano, Italy). The 3D cultures were incubated at 37 °C and 5% CO_2_ for seven days, with media changes every two days. Control conditions were represented by cells cultured on tissue culture plates (TCP) in cDMEM for the same culturing time.

Cell proliferation rate was evaluated after three and seven days from seeding with the MTT (methylthiazolyl-tetrazolium) based proliferation assay, performed according to the method of Denizot and Lang with minor modifications [22]. Briefly, samples were incubated for 3 h at 37 °C in 1 mL of 0.5 mg/mL MTT solution prepared in Phosphate Buffered Saline (PBS) (EuroClone). After removal of the MTT solution by pipette, 0.5 mL of 10% DMSO in isopropanol was added to extract the formazan in the samples for 30 min at 37 °C. For each sample, absorbance values at 570 nm were recorded in duplicate on 200 μL aliquots deposited in microwell plates using a multilabel plate reader (Victor 3, PerkinElmer Inc., Waltham, MA, USA).

LDH activity was measured using the Lactate Dehydrogenase Activity Assay Kit (Sigma-Aldrich, Saint Louis, MO, USA) according to the manufacturer's instructions. All conditions were tested in duplicate. The culture medium was reserved to determine extracellular LDH. The intracellular LDH was estimated after cells lysis with the assay buffer contained in the kit. All sampled were incubated with a supplied reaction mixture, resulting in a product whose absorbance was measured at 450 nm using a Victor 3 multilabel plate reader.

For SEM imaging, fibroblasts grown on samples for three and seven days were fixed in 2.5% glutaraldehyde in 0.1 M cacodylate buffer for 1 h, then progressively dehydrated in ethanol. All micrographs were obtained using a JSM JEOL 6490 SEM microscope (JEOL, Tokyo, Japan). The SEM analysis was performed at Centro di Analisi e Servizi Per la Certificazione (CEASC, University of Padova, Padova, Italy).

## 3. Results and Discussion

The formulation of W-D glass was conceived on the basis of a series of experiments concerning another method for the obtainment of highly porous glass-ceramics by direct foaming of precursor mixtures, based on preceramic polymers and reactive fillers [36,37]. More precisely, wollastonite–diopside (W-D) glass-ceramics were developed from silicone polymers filled with CaCO_3_ and Mg(OH)_2_. The use of preceramic polymers enabled the fabrication of highly porous foams by water release with the silicones still in the polymeric state at low temperature (300–350 °C), owing to the introduction of small amounts of hydrated salts. Hydrated salts corresponded to sodium tetraborate decahydrate (Na_2_O·2B_2_O_3_·10H_2_O) and to sodium phosphate dibasic heptahydrate (Na_2_HPO_4_·7H_2_O); both salts formed a liquid phase at the final firing temperature (1100 °C), at which wollastonite and diopside developed according to the reaction between CaO and MgO (from fillers) and silica (from the oxidative decomposition of silicones). According to the MTT assay, polymer-derived wollastonite-diopside foams showed promising results in terms of cell viability, LDH activity tests, as well as compressive strength [36,37].

**Figure 2:**
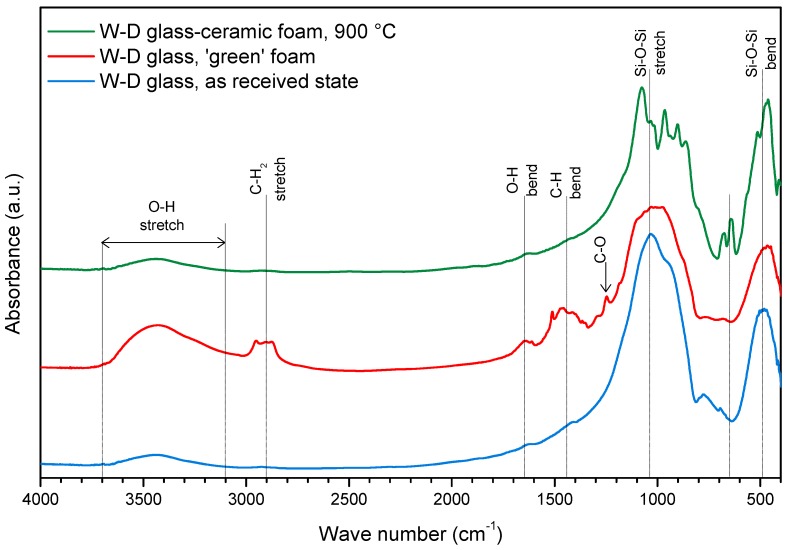
FTIR spectra of W-D glass, cured W-D glass foams, and W-D glass-ceramic foam after firing.

Previous experiments on the alkali activation of soda-lime glass cullet [32] have already demonstrated the ‘tobermorite-like’ nature of the formed gels. In particular, the hardening of glass suspensions was attributed to the formation of C–S–H compounds, owing to the appearance of a distinctive band in the FTIR spectra, in the 3000–3700 cm^−1^ range [25]. W-D glass after activation, direct foaming and drying (conditions of ‘green’ foam), as illustrated by Figure 2, exhibits the same band, assigned to stretching vibration of O–H groups. A weaker band around 1650 cm^−1^, assigned to deformation mode of O–H bond, also appears. In the as received state (lower plot in Figure 2), the broad band from 1290 to 900 cm^−1^ and the bands at 800 and 450 cm^−1^ could be ascribed to the stretching mode and with the rocking and bending of the Si–O–Si group, respectively [38,39,40]. In the alkali-activated state the same bands appear wider and flattened, likely due to the formation of more disordered bonding in the gel.

Compared to glass powders in the as received state, the alkali-activated material actually exhibited a slight formation of carbonate compounds (bands at 1420 and 1550 cm^−1^, from C–O bond), as well as bands (especially that centered at 2900 cm^−1^) attributable to C–H vibrations of the organic surfactant. The attribution to compounds subjected to thermal degradation is confirmed by the plot for a heat-treated sample (treatment at 900 °C—upper plot in Figure 2), very similar to that of the starting glass, except for the appearance of new defined bands at low wavenumber (1070, 1020, 960, 900, and 860 cm^−1^), attributed to the formation of crystalline silicates.

**Figure 3:**
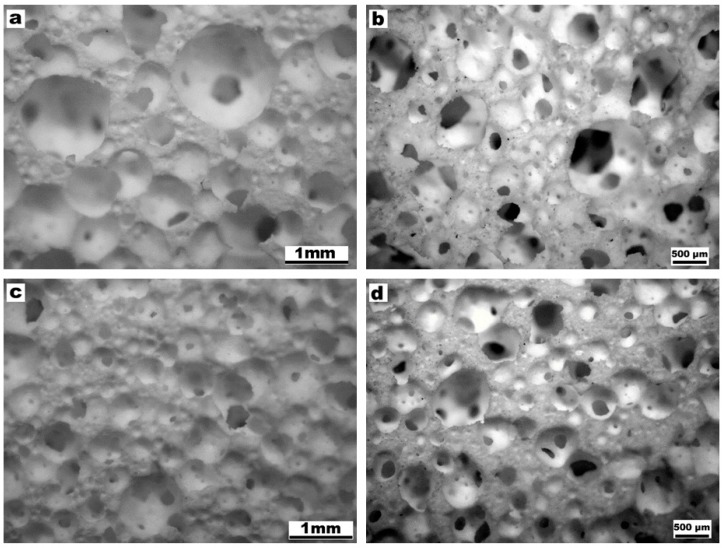
Microstructural and morphology details of W-D foams with different solid content of the starting suspensions, before and after firing at 1000 °C, respectively: (**a**,**b**) foams with 60 wt %; (**c**,**d**) foams with 65 wt %.

The successful low temperature gelification and foaming is illustrated by Figure 3. The alkali concentration, amount of surfactant and stirring speed were kept constant, while the solid content of the slurry was varied from 60 to 65 wt % to evaluate the change on the microstructure. An increase of glass content increased the ‘gelification ability’ of the mixtures; the consequent increase of viscosity of suspensions is consistent with a decreased pore size passing from 60 wt % solid content (Figure 3a) to 65 wt % solid content (Figure 3c). The different pore size was confirmed after firing (Figure 3b,d) and it was accompanied by a different overall porosity: as reported by Table 1 (discussed later), the higher solid content determined a decrease in the total porosity of about ~10 vol %.

**Figure 4:**
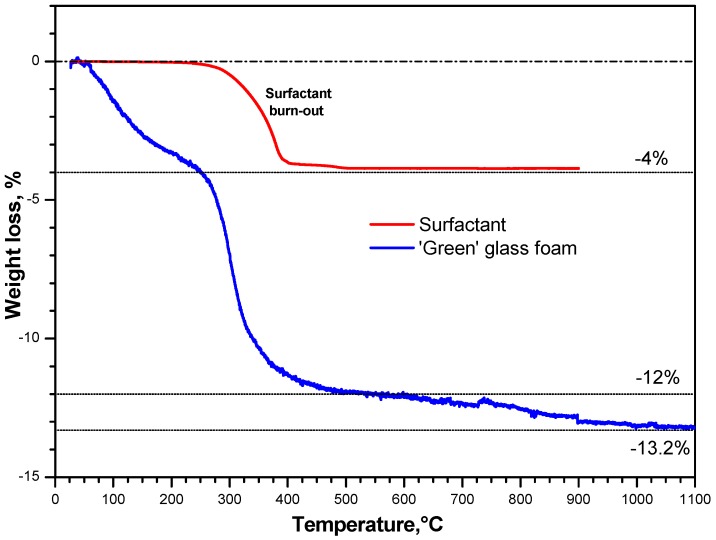
Thermo-gravimetric plot of alkali-activated W-D glass and surfactant.

The thermal evolution of the alkali-activated material is further explained by the thermo-gravimetric analysis reported in Figure 4. The plot for the gelified material is compared with that for pure Triton X-100; the latter plot is provided normalized according to the content of surfactant in the alkali-activated material, i.e., 4 wt %. It can be easily observed that the weight loss at 300–500 °C of the alkali-activated material cannot depend only on the burn-out of surfactant, corresponding to −12%; in addition, a slight weight loss is experienced above 600 °C, well above the temperature at which the surfactant decomposed completely.

In good agreement with what previously observed with soda-lime glass [32], the weight losses at low temperature (below 500 °C) can be ascribed, besides burn-out of surfactant, to physically absorbed water. The weight losses above 600 °C can be attributed to decomposition of hydrated compounds, with release of water from removal of –OH groups. In fact, C–S–H compounds are actually known to release water up to high temperatures [41].

**Figure 5:**
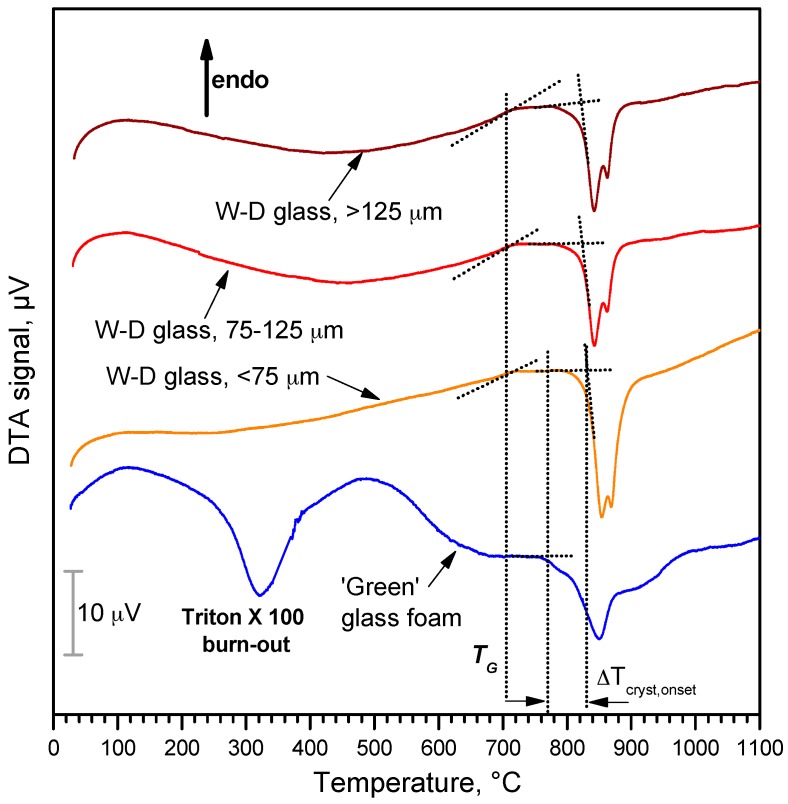
Differential thermal analysis of W-D glass (different particle sizes) and ‘green’ glass foam from alkali activation and direct foaming.

The differential thermal analysis plot in Figure 5 provides only a partial confirmation of the above-discussed phenomena. The plot for the alkali-activated material (lower plot) probably derives from many overlapping contributions; in our opinion, at low temperatures (<600 °C), there is overlapping between the exothermic effect of surfactant burn-out (centered at 300–320 °C, consistent with the onset of weight loss, for pure Triton X-100, in Figure 4) and endothermic effects of dehydration. At higher temperatures (>600 °C), corresponding to the final weight loss, the exothermic effects of glass crystallization become dominant. It is interesting to note that W-D glass is sensitive to surface crystallization, as demonstrated by the increased intensity of the crystallization peaks with decreasing particle size, as an effect of surface nucleation (both silicates are known to crystallize via this mechanism [42]), but it is also sensitive to the alkali activation. More precisely, the onset of crystallization is almost constant, with particle size, at about 830 °C, for pure glass, whereas the starting of exothermic effect is downshifted at about 770 °C for W-D glass after alkali activation. Alkali-rich surface gels reasonably transformed in an alkali-rich low viscosity liquid surrounding undissolved glass particles, promoted the ionic inter-diffusion and the crystallization (alkali rich glasses are known to feature lower activation energy for crystal growth [43]).

The enhanced crystallization for the alkali-activated material determined a substantial ‘freezing’ of the viscous flow sintering, so that samples sintered below 900 °C were particularly weak. Sintered at 900 °C, the samples actually featured some light grey areas, in a white matrix, reasonably due to some carbonaceous traces from the surfactant; on the contrary, samples sintered at 1000 °C were both mechanically consistent and homogeneously white.

**Figure 6:**
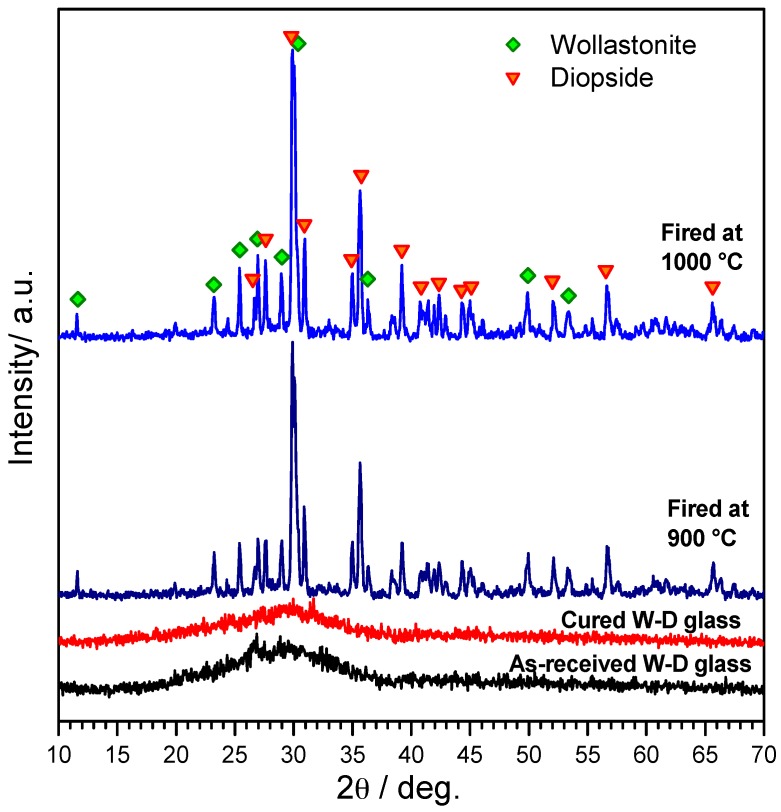
XRD patterns of W-D glass, cured W-D glass after demolding and W-D glass-ceramic foam after firing (heating rate: 5 °C/min).

The X-ray diffraction patterns, in Figure 6, further illustrate the evolution of samples, up to the firing at 900 and 1000 °C. The XRD pattern of alkali-activated materials provides additional evidence of the formation of gels (i.e., cured W-D glass foams after demoulding), owing to the slight shift of the amorphous halo at higher 2θ angles, a recognized proof the incorporation of network modifiers [44]. The patterns of the materials after firing at 900–1000 °C, on the other side, clearly demonstrate the obtainment of the desired phases, such as wollastonite (CaSiO_3_, PDF#27-0088) and diopside (actually a Mg-rich variant, Ca_0.89_Mg_1.11_Si_2_O_6_, PDF#87-698). As previously discussed, this combination of silicate phases is highly promising for the remarkable bioactivity of wollastonite and the mechanical strength of diopside [6,45,46,47,48].

Table 1 reports the physical and mechanical properties of glass-ceramic foams after firing at 1000 °C. It can be understood that, besides the solid load, also the heating rate may be seen as a ‘tuning factor’. Given the remarkable crystallization tendency, enhanced by the alkali activation, a low heating rate (2 °C/min) caused a limited viscous flow sintering, maximizing the content of both overall porosity and open porosity. In all cases, the compressive strength is substantial, exceeding 5 MPa for a sample still featuring abundant open porosity. The combination of good mechanical properties and interconnected porosity undoubtedly make the developed materials as promising candidates for bone regeneration (overall porosities of about 80 vol % are typically associated to compressive strength values not exceeding 2.5 MPa, in both sol-gel and melt-derived bioglass foams [21,49]).

**Figure 7:**
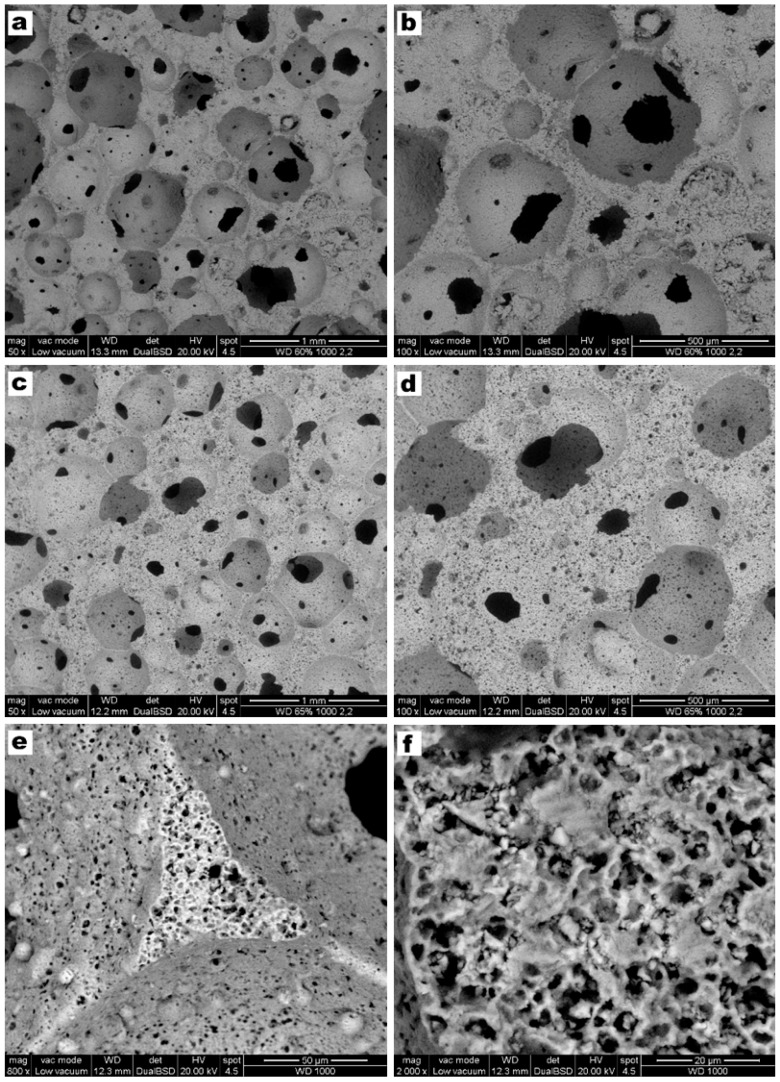
SEM images of W-D glass-ceramic foams with different solid content and after firing at 1000 °C (**a**,**b**) for foams with 60 wt % solid load; (**c**,**d**) for foams with 65 wt %; (**e**,**f**) high magnification details of cell struts.

Figure 7 collects microstructural details of porous W-D glass-ceramics from slurries with 60 and 65 wt % solids (heating rate: 2 °C/min), after firing at 1000 °C. The pore size was evaluated by means of image analysis using the Image J software [50], and expressed as the interquartile range. It is possible to observe the presence of open and interconnected cells, again, with different morphology depending on the total solid content, where slightly smaller pores at higher solid content can be noticed, going through 170–360 μm for the 60 wt % samples to 130–175 μm for the 65 wt % foams. This size pore reduction could be explained as an effect of increased viscosity with increasing solid content, in turn limiting cell coalescence. The SEM images of foams produced from 60 wt % slurry (see Figure 7a,b) show large openings between adjacent pores and thin struts. A higher solid load is accompanied by reduced interconnects (Figure 7c), but still well above the threshold (100 μm) [51] for good cell ingrowth and vascularization; the larger struts (Figure 7d) actually contain a multitude of micropores, so that the samples could be actually termed as featuring a ‘hierarchical porosity’. The micropores are more visible in the high magnification details (Figure 7e,f); some dense areas could be ascribed to former glass granules, surrounded by foamed material, reasonably derived from the thermal evolution of surface gels. The microporosity is undoubtedly favorable to cell attachment.

**Figure 8:**
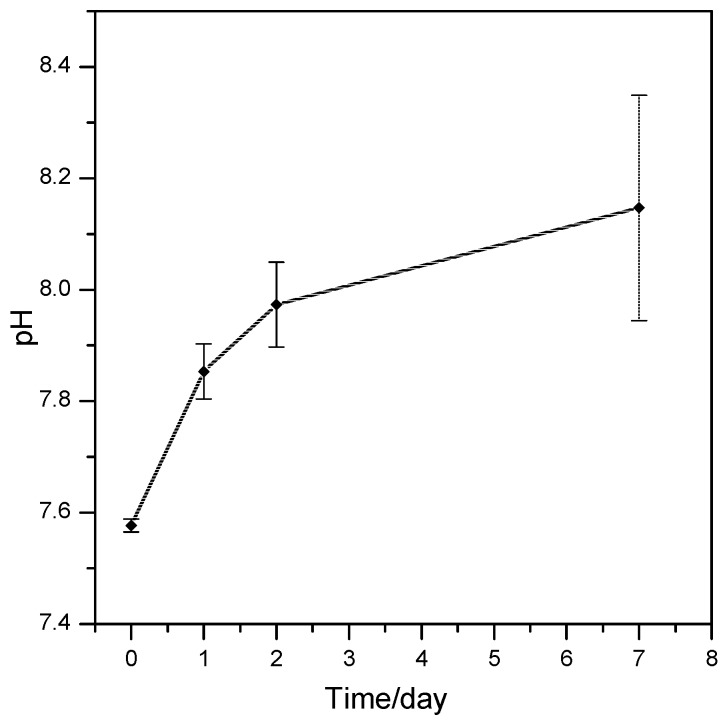
pH variation induced by the wollastonite-diopside glass-ceramic scaffolds over time.

The alkali surplus due to the activation process could lead to excessive leaching, if we consider that Na^+^ ions could be hosted only in the residual glass phase (both crystal phases do not contain alkali). The assessment of this issue is fundamental, since cells could be damaged by excessive pH levels or by fast pH variations. Figure 8 demonstrates that the pH of distilled water did not increase significantly over time, after immersion of glass-ceramic samples, as a consequence of limited release of alkaline or alkaline earth ions. More precisely, foamed samples with a weight of 75 mg were immersed in 50 mL distilled water and stored for seven days; the pH, measured in triplicate (at different time points for 1, 2, and 7 days, without refreshing) did not exceed 8.2. A detailed study of the ionic releases is beyond the aims of the present work; however, the limited alkalinity of solutions after immersion of samples was considered as a good starting point for cell viability studies.

**Figure 9:**
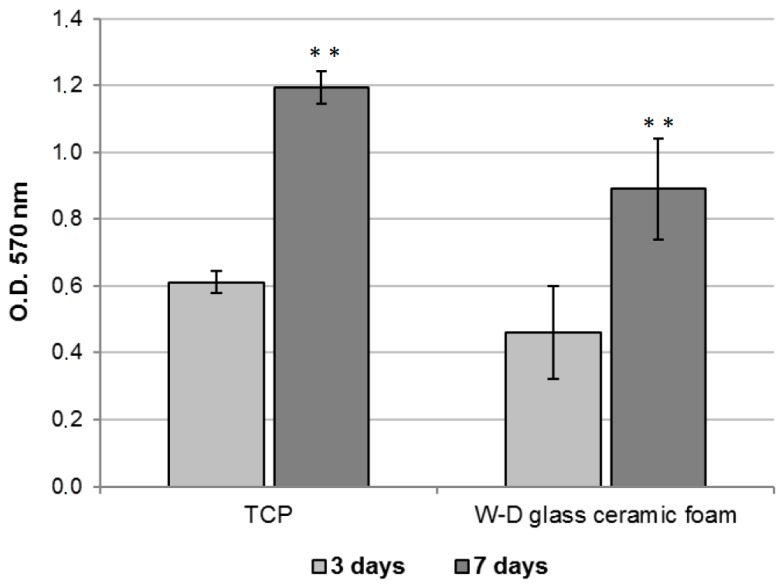
MTT assay of fibroblasts cultured on TCP (control condition) or on W-D glass-ceramic foam for three and seven days. Significant difference * (*p* < 0.05); ** (*p* < 0.01); *** (*p* < 0.001).

The MTT assay was performed in order to evaluate the viability of fibroblasts seeded onto W-D glass-ceramic foams for three and seven days. As shown in Figure 9, a significant increase in cell viability can be observed with culturing time, indicating the biocompatibility of the produced material. A similar trend in cell growth is evident in the control condition (TCP). This suggests that the viability of the fibroblasts was not affected by the formulation of the studied material.

**Figure 10:**
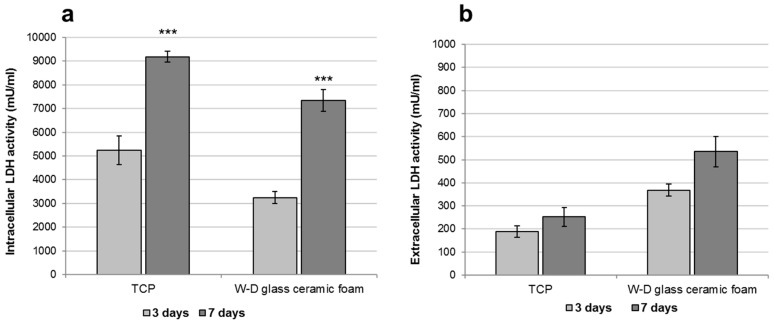
LDH activity assay of fibroblasts cultured on TCP (control condition) or on W-D glass-ceramic foam for three and seven days. (**a**) Intracellular LDH activity; (**b**) Extracellular LDH activity. Significant difference * (*p* < 0.05); ** (*p* < 0.01); *** (*p* < 0.001).

In order to assess the influence of the chemical composition of the material on fibroblast survival, the LDH activity assay was additionally performed. Figure 10a shows the intracellular LDH activity of the fibroblasts seeded onto W-D glass-ceramic foams for three and seven days. LDH activity was measured also on cells cultured in monolayer, which represented the control condition. The results suggest that cells seeded onto W-D glass-ceramic foams were able to produce metabolites, with significantly improved activity after seven days from seeding, as observed for fibroblasts grown onto TCP. The quantification of intracellular LDH displays the same tendency observed with the MTT assay. Culture medium was used to measure extracellular LDH activity. The graphs in Figure 10b show low levels of LDH activity in the extracellular culture medium both on cells cultured in monolayer and on the scaffold, thus indicating the absence of cytotoxicity of the W-D glass-ceramic foams.

**Figure 11:**
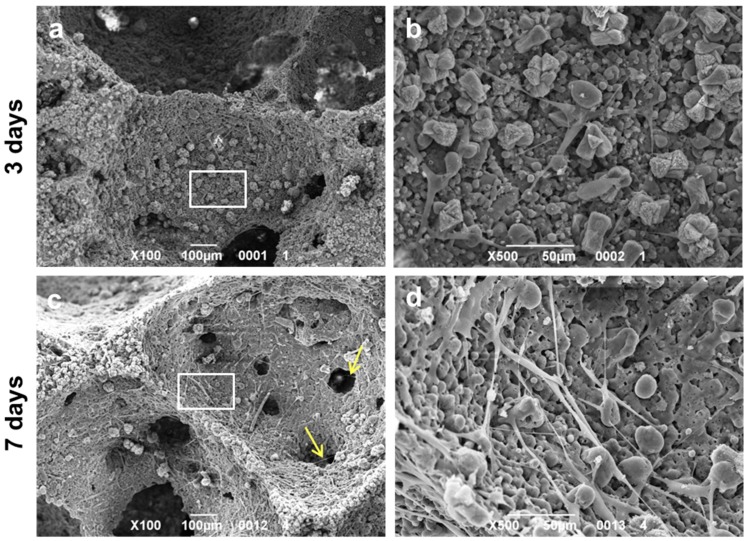
SEM images (100× and 500× magnification) of fibroblasts cultured on W-D glass-ceramic foam for (**a**,**b**) three and (**c**,**d**) seven days. Note that cells are able to migrate into the pores (**c**, yellow arrows) of the scaffold after seven days from seeding. The white boxes in (**a**,**c**) represent the areas shown at higher magnification in (**b**,**d**).

SEM images of the W-D glass-ceramic foams cultured with human fibroblasts are reported in Figure 11. After three days (Figure 11a,b) from seeding, fibroblasts were found to be spread and attached on the surface of the samples, showing their typical elongated morphology. After seven days (Figure 11c,d), cells had colonized the surface of the W-D glass-ceramic foams, displaying a better adhesion and proliferation but still showing elongated shapes. In addition, it is possible to note that at seven days from seeding, cells had not only completely populated the scaffold, but they had also infiltrated into its pores (yellow arrows in Figure 11c). All these observations further strengthen the evidence of the biocompatibility of the material.

## 4. Conclusions

We may conclude that:

- Highly porous wollastonite-diopside glass-ceramics can be easily obtained by low temperature ‘inorganic gel-casting’, followed by sintering with concurrent crystallization (sinter-crystallization); the crystallization limits the viscous flow, so that the microstructure in the green state is substantially maintained upon firing up to 1000 °C;
- The foaming relies on the progressive hardening of aqueous glass suspensions, after alkali activation; the gelification, owing to FTIR analysis, is consistent with the development of calcium silicate hydrates (C–S–H), later decomposed (with the firing treatment);
- The overall process (mechanical stirring of alkali activated suspensions—with the help of a surfactant, drying, firing with sinter-crystallization) has a great potential for the production of ‘hierarchically porous’ foams; the microstructure can be tuned operating on simple processing parameters such solid load, in suspensions, and firing conditions (e.g., heating rate);
- The developed glass-ceramics, according to MTT and LDH activity assays, with human fibroblasts, can be considered as biocompatible; forthcoming studies will focus on detailed studies of ionic releases and bioactivity.

## Figures and Tables

**Table 1:** Physical and mechanical properties of W-D glass-ceramic foams produced by different solid contents.

Solid Load	Heating Rate (°C/min), Up to 1000 °C	Bulk Density (g/cm^3^)	True Density (g/cm^3^)	Total Porosity (vol %)	Open Porosity (vol %)	Compressive Strength (MPa)
**60 wt %**	2 °C/min	0.29 ± 0.02	2.94 ± 0.01	90.6	90.1	3.50 ± 0.51
	5 °C/min	0.42 ± 0.05	2.95 ± 0.02	85.6	83.8	2.17 ± 0.10
**65 wt %**	2 °C/min	0.44 ± 0.03	2.97 ± 0.01	86.3	85.3	2.90 ± 0.50
	5 °C/min	0.53 ± 0.04	2.95 ± 0.01	81.9	81.1	5.30 ± 0.74
